# No evidence of bovine leukemia virus proviral DNA and antibodies in human specimens from Japan

**DOI:** 10.1186/s12977-022-00592-6

**Published:** 2022-05-18

**Authors:** Meripet Polat Yamanaka, Susumu Saito, Yukiko Hara, Ryosuke Matsuura, Shin-nosuke Takeshima, Kazuyoshi Hosomichi, Yasunobu Matsumoto, Rika A. Furuta, Masami Takei, Yoko Aida

**Affiliations:** 1 Laboratory of Global Infectious Diseases Control Science, Department of Global Agricultural Sciences, Graduate School of Agricultural and Life Sciences, The University of Tokyo, 1-1-1 Yayoi, Bunkyo-ku, Tokyo, 113-8657 Japan; 2 Viral Infectious Diseases Unit, RIKEN, Saitama, 351-0198 Japan; 3 Division of Hematology and Rheumatology, Department of Medicine, Nihon University School of Medicine, Tokyo, 173-8610 Japan; 4 Division of Department of Breast and Endocrine Surgery, Department of Surgery, Nihon University School of Medicine, Tokyo, 173-8610 Japan; 5 Department of Food and Nutrition, Jumonji University, Saitama, 352-8510 Japan; 6 Department of Bioinformatics and Genomics, Graduate School of Medical Sciences, Kanazawa University, Ishikawa, 920-8640 Japan; 7 Laboratory of Global Animal Resource Science, Graduate School of Agricultural and Life Sciences, The University of Tokyo, Tokyo, 113-8657 Japan; 8 Central Blood Institute, Blood Service Headquarters, Japanese Red Cross Society, Tokyo, 135-8521 Japan

**Keywords:** Bovine leukemia virus (BLV), Japanese human blood, Japanese breast cancers, Japanese human sera, PCR, Antibody detection

## Abstract

**Background:**

The potential risk and association of bovine leukemia virus (BLV) with human remains controversial as it has been reported to be both positive and negative in human breast cancer and blood samples. Therefore, establishing the presence of BLV in comprehensive human clinical samples in different geographical locations is essential.

**Result:**

In this study, we examined the presence of BLV proviral DNA in human blood and breast cancer tissue specimens from Japan. PCR analysis of BLV provirus in 97 Japanese human blood samples and 23 breast cancer tissues showed negative result for all samples tested using long-fragment PCR and highly-sensitive short-fragment PCR amplification. No IgG and IgM antibodies were detected in any of the 97 human serum samples using BLV gp51 and p24 indirect ELISA test. Western blot analysis also showed negative result for IgG and IgM antibodies in all tested human serum samples.

**Conclusion:**

Our results indicate that Japanese human specimens including 97 human blood, 23 breast cancer tissues, and 97 serum samples were negative for BLV.

**Supplementary Information:**

The online version contains supplementary material available at 10.1186/s12977-022-00592-6.

## Background

Bovine leukemia virus (BLV) is the causative agent of enzootic bovine leucosis (EBL), a form of B cell lymphoma in cattle, which is closely related to human T-cell leukemia viruses (HTLVs) [1]. BLV has spread worldwide and causes serious problems in the cattle industry [2]. Transmission of BLV within a herd can occur primarily through contact with bodily fluids containing infected lymphocytes via blood-sucking insects or through iatrogenic procedures, including repeated use of individual needles, syringes, rectal palpation gloves, and dehorners [3]. Initially, BLV infects various cell populations, but only BLV-infected B cells can induce the mono- or oligo-clonal expansion of origin of B-cells after a long latency period [1]. Eventually, BLV-induced B-cell lymphoma can occur in different organs and tissues, leading to a series of defects that cause mortality in the animal [3]. The majority of BLV-infected cattle are asymptomatic carriers of the virus, but less than 5% of infected cattle develop lymphoma [1]. This means that most infected cattle remain healthy, produce optimal amounts of milk, and are therefore, not culled from the herd. Therefore, BLV-infected B cells circulate through the blood of infected cattle and are present in beef [4], and infectious BLV-infected cell are also present in the milk of infected dairy cows [5–7]. This can be a means of transmission from cows to human. However, whether BLV is transmissible in humans remains unclear.

BLV naturally infects cattle, water buffalo, yaks [8] and alpaca [9–11], but can experimentally infect various other species, including sheep [12–15], rats [16], rabbits [16–18], pigs [19], chickens [19, 20] and goats [21] as well as several cell lines including human cells such as Hela cells and kidney 293T cells [22, 23]. Human cells of neural origin are also highly susceptible to BLV infection [16].

A few studies on the development of new and more sensitive methods have revealed re-emerging concern regarding the possible connection of BLV and human disease. An initial study by Buehring et al. reported the detection of antibodies against BLV p24 protein in 74% of the tested human sera [24]. A decade later, the same group also detected the BLV proviral gene *tax* in 44% (97/219) of human breast cancer tissue samples using in situ polymerase chain reaction (PCR), and 6% (12/215) of these samples showed the presence of viral p24 protein by immunohistochemistry [25]. Since then, either the BLV provirus (*gag*, *env*, *tax*, long terminal repeat (LTR)) and/or antibodies reacting to BLV antigens (p24 and gp51) have been detected in a wide range of geographical regions such as the United States [26–29], Australia [30, 31], Columbia [32], Brazil [29, 33] and Iran [34], in human breast tissue (healthy, benign, pre-malignant, or malignant), as well as in human blood and serum samples. These results indicate a high odds ratio of association between the presence of BLV DNA and breast cancer through screening the presence of BLV in the breast tissue of women with cancer and that of women without cancer using in situ PCR [26, 27, 29, 32]. BLV proviral DNA has also been detected in human squamous cell lung carcinoma [35].

In contrast, although studies concerning the possible zoonotic potential of BLV started before the discovery of BLV and at subsequent periods in different geographical locations [36, 37], none of the epidemiological and serological studies carried out in the 1970s showed a direct and clear connection with BLV [36, 38] and could not find any individual with antibodies to BLV, even though most examined people were naturally exposed to the virus [6, 37, 39–42]. Further, a subsequent study aiming to investigate any potential role of BLV in human leukemia demonstrated the absence of BLV-specific sequences in 157 cases of childhood acute lymphoblastic leukemia or non-Hodgkin’s lymphoma and 136 controls in the United States [43]. In China, 91 breast cancer tissues and 160 blood samples were negative for antibodies against BLV [44]. In Korea, BLV proviral sequences were found to be absence in 517 cases of human leukemia and 162 cases of lung cancer [45], as well as in whole genome sequencing of 51 breast cancers [46]. Further, in Japan, BLV proviral DNA was not detected in human blood cell lines and cancer cell lines, even with a very sensitive PCR method capable of detecting even one copy of BLV in the positive control [47]. Therefore, it was believed that BLV was not transmissible to human.

As mentioned above, despite extensive study and expanded knowledge about the epidemiology and biology of BLV and its possible role in human malignancies, the potential risk and association of BLV with humans continues to be controversial and remain unresolved. Confirming the correlation of BLV with human is thus of great importance as BLV is distributed worldwide in cattle, and there are several cases in which humans can be exposed. Therefore, additional research is essential to establish the presence of BLV in increasing numbers of human clinical samples from different geographical locations compared to those in previous studies. In Japan, a current nationwide serological survey of BLV infection showed a high BLV sero-prevalence (40.9% in dairy cows, 28.6% in breeding cattle, and 68.1% at the herd level) and an increase in the incidence of EBL [48, 49]. In our previous study, human blood cell lines and cancer cell lines from Japanese people were detected as BLV negative through PCR amplification only [47]. In the current study, we expanded on the previous study to include the screening of Japanese human specimens including blood, breast cancer tissue, and serum samples using both DNA-based PCR methods and antibody-based serological tests to better understand the association between BLV infection and human cancer. Therefore, we first determined whether BLV proviral DNA is present in human donated blood and in breast cancer tissue specimens from Japan. We then assessed the presence of BLV reactive antibodies in the human sera from volunteer blood donors who were negative for ten infectious diseases, including HTLV and human immunodeficiency virus (HIV).

## Result

### BLV proviral DNA detection in human samples by long-fragment amplification of BLV provirus

**Fig. 1:**
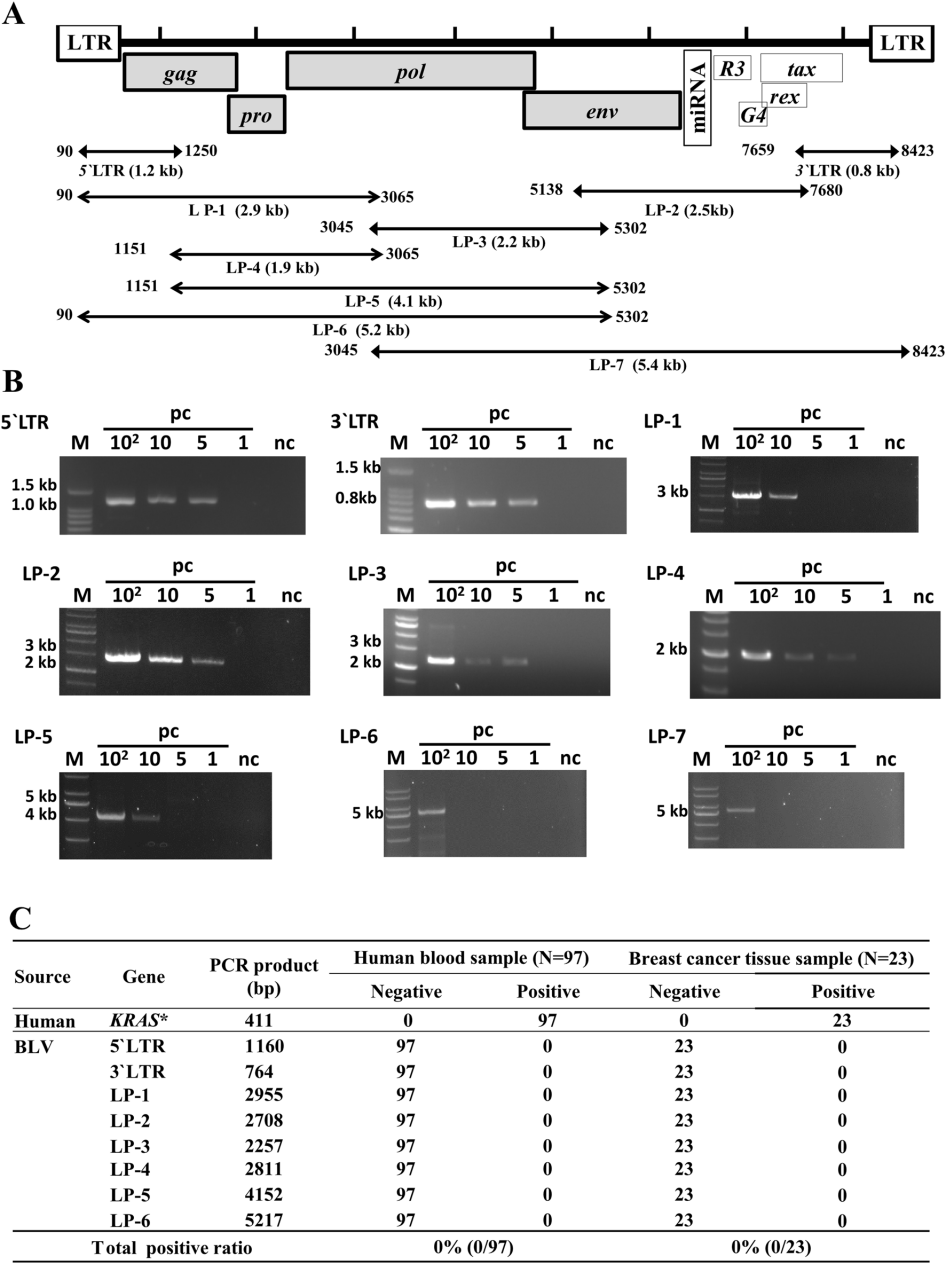
Detection of BLV provirus in human blood and breast cancer tissue samples by long- fragment PCR. **A** Schematic presentation of PCR amplification of larger fragment of BLV proviral gene in human samples. The Numbers on the two sides of the double arrow lines indicate the beginning and ending of the fragment amplified by PCR. The number under the double arrow line shows PCR ID and the length of each PCR product. **B** Determination of long PCR (LP) sensitivity. Positive control (pc) FLK-BLV DNA were diluted to obtain BLV copy numbers of 100, 10, 5 and 1 copy in each PCR reaction. Distilled water was used as negative control (nc). M indicates molecular weight marker (5`LTR and 3`LTR: 100 bp DNA ladder (MIXELL Inc, Hiroshima, Japan); LP-1 ~ LP-6: 1 kb DNA ladder). **C** Summary of PCR amplification of long-fragments of BLV genome. *The quality of DNA samples extracted from human samples were assessed by PCR amplification of human *KRAS* gene

Previously, BLV proviral DNA was independently identified in breast tissues, lung carcinoma and blood samples [24–26, 28, 30, 31, 34, 50]. However, only one fragment was detected, but the full-length BLV genome was not detected in human tissues. Therefore, to investigate the presence of BLV in humans, we amplified the long region of the BLV genome, as shown in Fig. 1A. We determined the PCR sensitivity using DNA from FLK-BLV cells, which are permanently infected with BLV, as a template. FLK-BLV DNA was diluted to 100, 10, 5 and 1 BLV provirus copies/reaction and measured using droplet digital PCR. PCR products of the 5LTR (1.2 kb), 3’LTR (0.8 kb), LP-1 (2.9 kb), LP-2 (2.7 kb), LP-3 (2.2 kb), LP-4 (2.8 kb), LP-5 (4.1 kb), LP-6 (5.2 kb), and LP-7 (5.4 kb) fragments were analyzed (Fig. 1B). These PCR products were sequenced, and the sequences of all six regions were found to be identical to those of the FLK-BLV subclone pBLV913 (NCBI accession number: EF600696). As shown in Fig. 1B, even though BLV provirus were amplified from the DNA template of BLV with 5 copies/reaction in five (5′LTR, 3′LTR, LP-2, LP-2 and LP-4) of nine PCR sets, all of nine targets were more stably amplified by PCR using the DNA template of BLV copies as equal to and/or more than 10 copies/reaction (BLV proviral copy ≥ 10).

Next, we investigated the presence of BLV provirus in DNA extracted from 97 donated human blood samples and 23 breast cancer tissue samples from Japan. Before performing this experiment, the quality of genomic DNA was assessed by amplification of codon 12/13 region of human *KRAS* proto-oncogene. A 411-bp fragment of the *KRAS* gene was detected in all samples (Fig. 1C). Therefore, all the human DNAs were screened for BLV provirus amplification using long PCR, and the PCR products were separated by electrophoresis on a 3% agarose gel. Each human sample was tested twice or three times. As summarized in Fig. 1C, none of the PCR sets successfully amplified larger fragments of BLV provirus in all 97 human blood samples and 23 breast cancer tissue samples.

### BLV proviral DNA detection in human samples by short-fragment amplification of BLV provirus

**Fig. 2:**
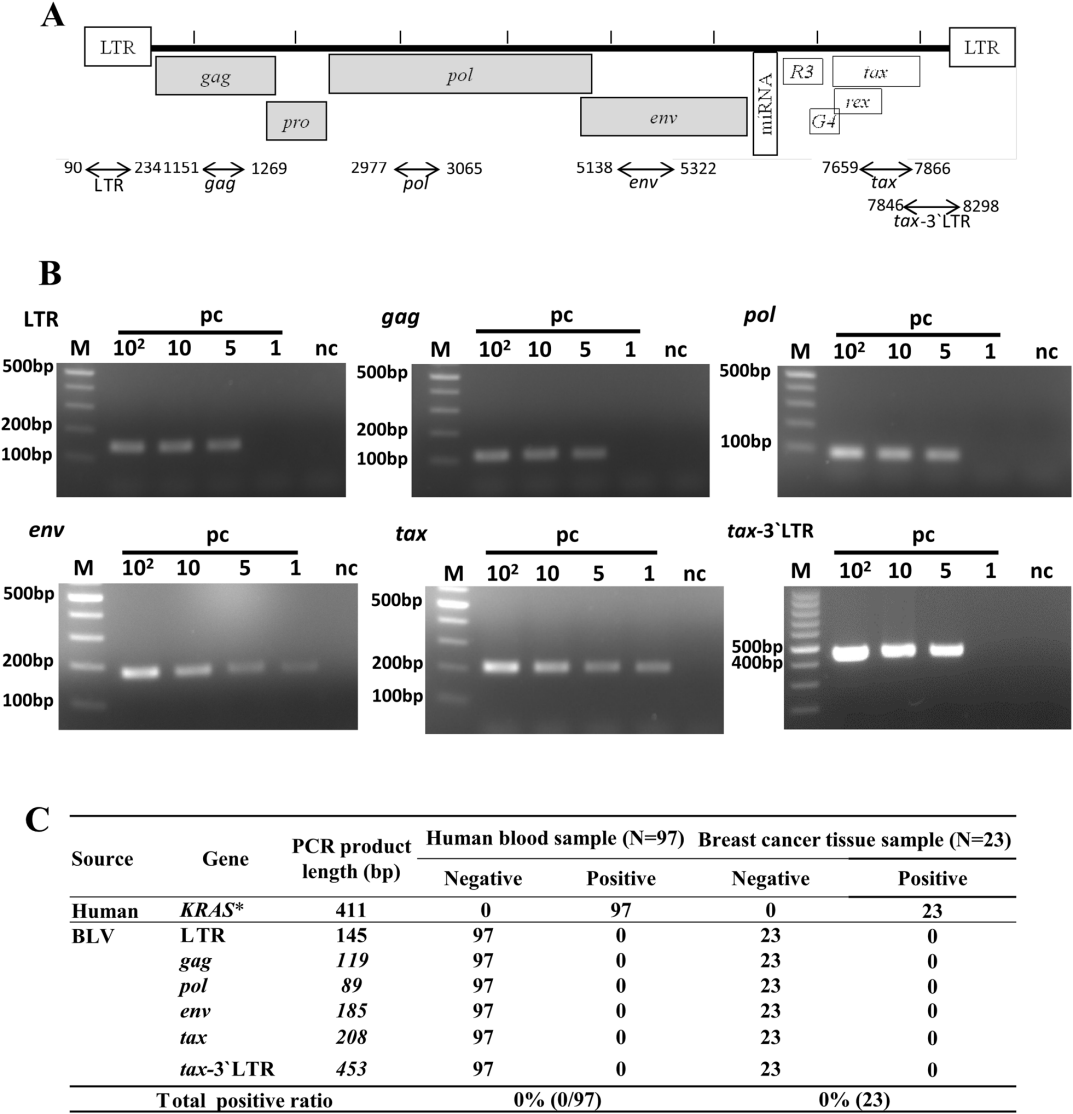
Detection of BLV provirus in human blood and breast cancer tissue samples by short-fragment PCR. **A** Schematic representation of PCR amplification of short region of proviral gene in human samples. Numbers on the two sides of double arrow lines indicate the beginning and ending of the fragment amplified by PCR. The number under the double arrow line indicates the BLV proviral gene (LTR; *gag*; *pol*; *env*; *tax* and *tax-*3`LTR). **B** Electrophoresis result of PCR products. Each sample was tested in triplicate. Positive control (pc) DNA obtained from FLK-BLV cells was adjusted to BLV copy numbers of 100, 10, 5 and 1 copy in each PCR and amplified in duplicated. Distilled water was used as the negative control (nc). M indicates molecular weight marker (100 bp DNA ladder, (MIXELL Inc, Hiroshima, Japan)). **C** Summary of BVL detection in human samples by short range PCR. *The quality of DNA samples extracted from human samples were assessed by PCR amplification of human *KRAS* gene

All human samples were then subjected to highly sensitive PCR method using six primer sets to amplify short regions of the BLV genome (Fig. 2A). FLK-BLV DNA was diluted to BLV copy numbers of 100, 10, 5 and 1 per PCR and the PCR products were separated by electrophoresis on a 3.0% agarose gel. Predictably, the PCR products were detected from LTR (145 bp), *gag* (119 bp), *pol* (89 bp), *env* (185 bp), *tax* (208 bp), and *tax*-3 LTR (453 bp). These PCR products were sequenced, and then the sequences of all six regions were found to be identical to those of FLK-BLV subclone pBLV913 (NCBI accession number: EF600696). As shown in Fig. 2B, five copies of BLV in the template were constantly amplified in all six target regions, and BLV *env* and *tax* genes showed amplification of one copy of BLV in the template DNA. This result indicates the high sensitivity of short-fragment PCR compared to long-fragment PCR.

Next, using this highly sensitive PCR, we examined the BLV provirus in DNAs extracted from 97 donated human blood samples and 23 breast cancer tissue samples from Japan. Each human sample was tested two to three times. However, none of the study subjects were found to be positive for any of the six BLV genome regions tested in blood [0% (0/97)] and breast cancer tissue samples [0% (0/23)] (Fig. 2C).

### Detection of anti-BLV antibodies in human serum samples from Japan using enzyme-linked immunosorbent assay (ELISA)

Here, we hypothesized that if BLV was present in human, the host immune system would produce BLV antigen-related antibodies. Indeed, previous studies have demonstrated the detection of antibodies to BLV p24 protein in 74% of human sera tested [24]. Therefore, we performed ELISA test with the aim of detecting antibodies against BLV in sera from the same blood, which yielded negative results in the short- and long-fragment PCR amplification. These sera were separated from the donated blood samples for testing infectious diseases and biochemical tests and were guaranteed to have no cross-reactivity with other human infectious diseases, including HTLV and HIV, as shown in Table 1. In particular, because BLV is closely related to HTLVs [1] and some cross-reactivity has been shown between BLV and HTLV-I antigens [51], we performed a HTLV-I antibody test using the ELISA system to exclude serums that had the non-specific reaction with the BLV antigen, and clearly showed that all sera samples used in this study have no cross-reactivity between BLV and HTLV-I antigens.

**Table 1:** Summary of infectious disease testing of blood samples used in this study

Target testing	Method	Result
Hepatitis B surface (HBs) antigen testing	CLEIA	Negative
Hepatitis B core (HBc) antibody testing	CLEIA	Negative
HB virus (HBV) DNA nucleic acid testing	PCR	Negative
Hepatitis C virus (HCV) antibody testing	CLEIA	Negative
HCV DNA nucleic acid testing	PCR	Negative
Human immunodeficiency virus type-1, 2 (HIV-1, 2) antibody testing	CLEIA	Negative
HIV-1, 2 DNA nucleic acid testing	PCR	Negative
Human T-leukemia virus type 1 (HTLV-I) antibody testing	CLEIA	Negative
Syphilis treponemal antibody testing	CLEIA	Negative
Human parvovirus B19 antigen testing	CLEIA	Negative

CLEIA: Chemiluminescence enzyme immunoassay; PCR: Polymerase chain reaction

Before performing the BLV gp51 and p24 ELISA, we first validated whether the anti-human secondary antibodies (horseradish peroxidase (HRP)-conjugated goat anti-human IgG and IgM) used in our study are actively working or not. We thus selected the HTLV I/II antibody ELISA kit and performed ELISA using HRP-conjugated goat anti-human IgG and IgM in parallel with the secondary antibody provided in the kit. Two HTLV-I/II positive sera reacted strongly with high levels of coated HTLV I/II antigen (optical density (OD) value = 1.7–2.8 in two experiment), whereas two HTLV-I/II-negative sera did not react (OD value = 0.06–0.07 in two experiment) (Table 2). These results were similar to those of the HTLV I/II ELISA kit using the secondary antibody provided in the kit, indicating that the HRP-conjugated goat anti-human IgG and IgM are working properly. Next, we performed BLV gp51 and p24 ELISAs in BLV gp51 and p24 antigen-coated plates using 50-fold-diluted human serum specimens as primary antibodies, followed by either HRP-conjugated goat anti-human IgG or HRP-conjugated goat anti-human IgM as secondary antibodies (Table 2). To determine the validity of the experimental procedure, we concomitantly performed the experiment with samples from two BLV-infected cattle and one cattle without BLV infection, as positive and negative controls, respectively, followed by incubation with HRP-conjugated anti-bovine IgG as secondary antibody. Two 200-fold-diluted bovine sera from BLV-infected cattle with lymphoma were found to react strongly with coated gp51 (OD value = 0.8–1.2 in triplicate) and p24 antigens (OD values = 1.4–3.2, in triplicate), but 200-fold-diluted BLV-negative serum did not show any reaction with gp51 (OD value = 0.07–0.1 in triplicate) or p24 antigen (OD values = 0.2 in triplicate) (Table 2).

**Table 2:** Summary of anti-BLV antibody detection in human serum by ELISA

2nd Antibody	Human sera specimens		Control experiment					
			Bovine sera				Human sera	
	BLV gp51^a^ ELISA (N = 97)	BLV p24^a^ ELISA (N = 97)	BLV gp51 ELISA^b^		BLV p24 ELISA^b^		HTLV-I/II ELISA^c^	
			BLV (+) (N = 2)	BLV (−) (N = 1)	BLV (+) (N = 2)	BLV (−) (N = 1)	HTLV (+)	HTLV (−)
Goat anti-human IgG (HRP)	0/97	0/97	0/2	0/1	0/2	0/1	2/2	0/2
Goat anti-human IgM (HRP)	0/97	0/97	0/2	0/1	0/2	0/1	2/2	0/2
Rabbit anti-bovine IgG (HRP)	NT	NT	2/2	0/1	2/2	0/1	0/2	0/2

^a^BLV gp51 and p24 ELISAs in BLV gp51 and p24 antigens coated plates was performed using 50-fold-diluted human serum specimens as primary antibody following either HRP-conjugated goat anti-human IgG or HRP-conjugated goat anti-human IgM as secondary antibody. NT indicates not tested

^b^BLV gp51 and p24 ELISAs was performed using two BLV-infected cattle and one cattle with no BLV infection, as positive and negative controls, respectively, followed by HRP-conjugated anti-bovine IgG, as second antibody to check the validity of the experiment procedure

^c^HTLV I/II antibody ELISA was performed using human HTLV I/II positive antibody and negative antibody, followed by HRP-conjugated goat anti-human IgG and IgM in replacement with the second antibody in the kit to validate whether anti-human second antibodies HRP-conjugated goat anti-human IgG and IgM used in our study are actively working or not

In contrast, all 97 human serum specimens were negative for both of the antibody isotypes, IgG and IgM, against BLV gp51 and p24 with OD values ranging from 0.01 to 0.23 for anti-gp51 and from 0.01 to 0.05 for anti-p24. Thus, the ELISA showed that all 97 human sera samples from Japan had no antibodies reactive with BLV antigens (Table 2).

### Detection of antibodies to BLV in human samples from Japan as determined by western blotting

**Fig. 3:**
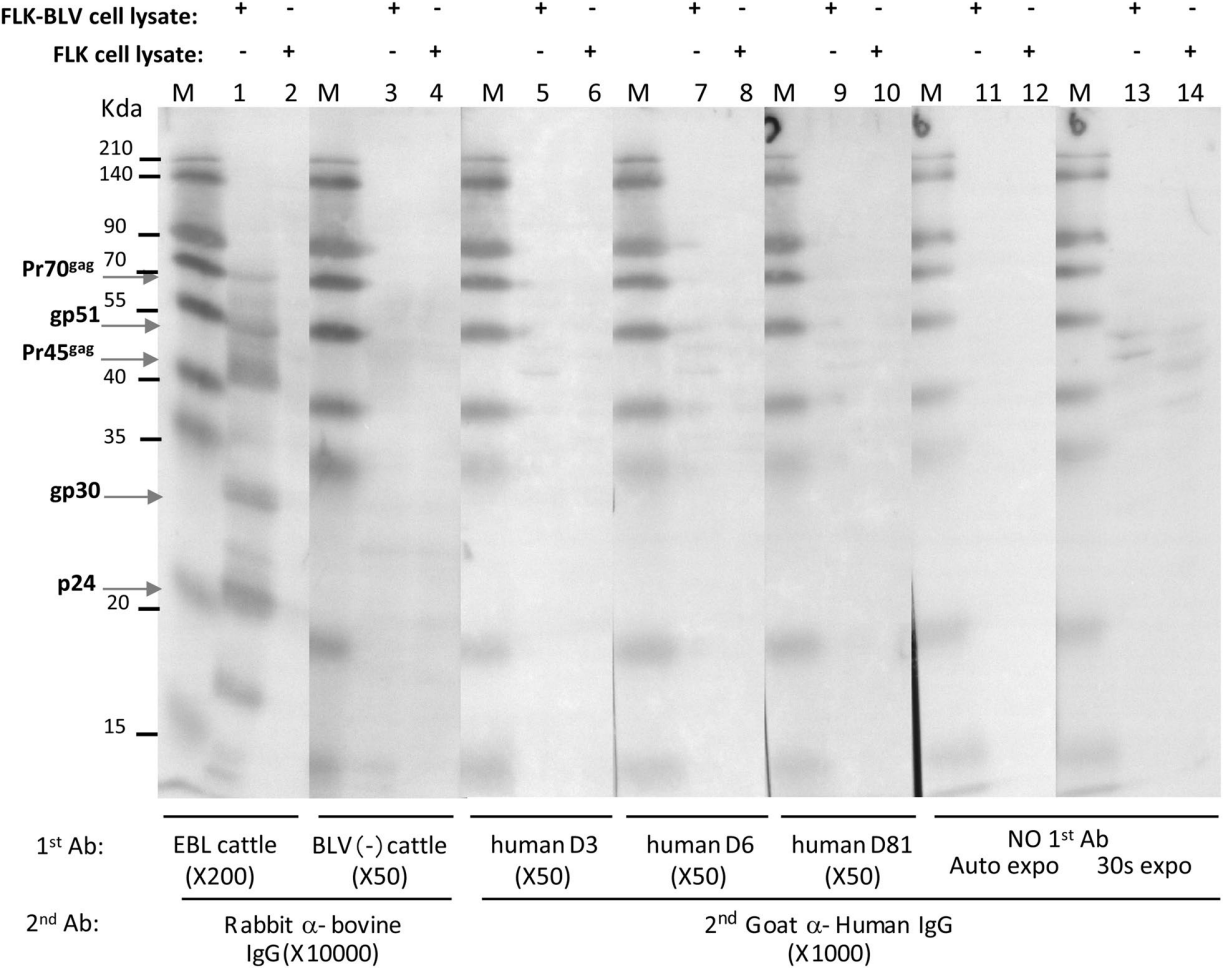
Western blot analysis to test the anti-BLV reactivity of human serum. Human serum samples were used as primary antibody. M indicates protein molecular weight marker; + indicates FLK-BLV cell lysate applied on the SDS-PAGE; − indicates BLV-free FLK cell lysate applied on 12% SDS-PAGE; EBL cattle indicate serum obtained from BLV-infected with EBL; BLV (−) cattle indicates serum from cattle without BLV infection; No 1st Ab control indicates that membrane was incubated with 5% skim milk in PBS without any primary antibody

We also performed western blotting analysis to test 31 human serum samples for anti-BLV antibody screening. Sera from BLV-infected cattle with EBL and from BLV-negative cattle were used as positive and negative control, respectively, to check assay. An only-secondary antibody (no primary antibody) control was used to check for non-specific binding of the secondary antibody with BLV antigens. Bands corresponding to structural proteins, such as p24, gp30, Pr45^gag^, gp51 and Pr70^gag^, were specifically detected in analyses of the permanently BLV-infected FLK-BLV cells (Fig. 3 lane 1), but not in the BLV-free FLK cells (Fig. 3 lane 2), by western blotting analysis using serum from BLV-infected cattle with EBL. In contrast, no specific bands were detected with sera from uninfected cattle (lanes 3 and 4). Although all samples were run triplicate, only the bovine test positive control showed reactivity of antibodies against BLV antigens; none of the human samples showed either IgG or IgM reactivity with BLV antigens. However, some non-specific bands were observed in 17 of the 31 human sera samples (Fig. 3 lanes 5, 7 and 9), which were further explained by non-specific binding of secondary antibody (goat-anti-human IgG) corresponding with bands through longer exposure of only the secondary antibody control without the primary antibody (Fig. 3 lanes 13 and 14). The present data demonstrate that the 31 human sera from Japan used for western blotting were positive for 0% (0/31), consistent with the ELISA results.

## Discussion

As the association between BLV and human cancer remains controversial, additional studies are needed to confirm the presence of BLV in comprehensive human clinical samples from different geographical locations. In this study, we investigated the presence of BLV in clinical samples from Japan including human blood and breast cancer tissue samples; our result led to the following two conclusions. First, our effort to amplify both short- and longer-fragments of BLV provirus in 97 human blood and 23 breast cancer tissue samples from Japan resulted in non-amplification. This result is consistent with our previous study on BLV absence in Japanese human blood cell lines and cancer cell lines [47] as well as with a Chinese study [44], and a Korean report [45]. However, our results were contrary to previous reports indicating the presence of BLV proviral DNA fragments in human blood and breast tissues from different geographical locations, such as USA [24–28], Australia [30, 31], Iran [34] and Brazil [29, 33]. These controversial conclusions from different studies could be due to the difference in sensitivity of the screening methods applied, different specimen types used, and the experiment subjects from different human populations in each study. Second, serological tests (ELISA) for antibodies (IgM and IgG) against BLV antigen showed the absence of both IgM and IgG against BLV antigens in 97 human sera from Japan. The ELISA results were further confirmed using western blot analysis indicating that neither IgM nor IgG antibodies interacting with BLV were present in the tested samples. Thus, our immunological tests showed that all human serum samples from Japan had no antibodies reactive with BLV antigens. However, this is contrary to a previous report on the presence of antibodies against BLV in human serum [24]. Taken together, our results clearly showed no evidence of BLV proviral DNA in human donated blood and breast cancer, and of antibodies reactive in human sera from Japan.

During the BLV-induced leukemia progression, BLV proviral genes coding for polymerase, capsid, and envelop proteins become increasingly deleted in BLV-infected cattle to escape from the host immune response, while BLV still retains the LTR region and *tax* gene, which carry oncogenic potential [9, 52, 53]; it is not clear whether BLV can cause the same phenomenon in human body, considering that all the blood donors are healthy. Further, although BLV proviral DNA has been independently identified in breast tissues [25–27, 29, 30, 33, 34, 54, 55], lung carcinoma [35], and blood [28, 34], only a fragment and not the full-length BLV genome has been detected in human tissues. Therefore, in current study, we tried to amplify nine long fragments of BLV provirus as well as six short fragments corresponding of different proviral genes in human blood and breast cancer tissue samples to detect any possibly BLV-infected target specimens. Even though long-fragment PCR showed stably amplification of 10 or more copies of BLV, short-fragment PCR results are highly sensitive, as an average five copies of BLV provirus in template, and the *env* and *tax* genes in one copy of BLV provirus in DNA template can be amplified. The high sensitivity of short-fragment PCR is consistent with that reported in our previous work [47]. Both short- and long-fragment PCR amplification of BLV provirus in human samples showed that none of the human blood and tissue samples were BLV positive, even in a single target region, indicating that the Japanese human blood and breast cancer tissue samples screened were negative for BLV.

To evaluate and confirm the specificity of the prime pairs in this study, we compared sequences of all primers, including primers in our previous publications and the new primers designed in this study, against sequences in nucleotide database using NCBI BLAST. Comparisons of E values indicate high homology only with BLV (E = 0.038 ~ 0.59) and low homology with human genome, including endogenous retrovirus and other retroviruses (E = 1.3 ~ 898). E values ≤ 1.00 indicate a low probability of random chance similarity and therefore a high specificity as a primer match for the targeted BLV sequence; E values > 1.00 indicate a high probability that sequences being compared are similar because of random chance [28]. Therefore, the BLV primers used in this study were highly specific for amplifying the BLV proviral genome and has a very low probability of amplifying other retrovirus and human genome sequences, including endogenous retroviruses. The sensitivity of PCR was confirmed by the amplification of BLV provirus as low as one copy number in PCR (short-fragment PCR). All experimental procedures, including PCR amplification, ELISA and western blot analysis of each sample, were performed at least three times at different time, with the same result, indicating the reproducibility of our study.

The results of the antibody detection by ELISA (BLV gp51 & p24 indirect ELISA) were consistent with those of western blotting analysis. Both assays showed that human samples were negative for both IgM and IgG antibodies against the BLV. It is contrary to a previous report indicating that humans have antibodies reactive to BLV in human serum [24, 28]. The presence of antibodies to particular viruses in human serum is generally interpreted as an indicator of a present or past infection with a virus, whereas absence of antibodies suggests lack of infection [56]. The possible explanations for the absence of anti-BLV antibodies in human serum are that BLV may not be infectious to humans or the tested individuals were not exposed to BLV, or the levels of antibodies were extremely low, being undetectable. For example, our ELISA systems showed that in a series of dilutions of cattle serum E4 (the European Community’s reference serum), E4 was reacted at 1:12,800 [57] and the cut-off point for the S/P value of the p24 ELISA and the gp51 ELISA was 0.40 and 0.30, respectively. Another possible explanation is that humans are infected but do not develop any immune response against BLV.

BLV is transmitted to cattle primarily by direct exposure to bodily fluids containing infected cells, such as blood and milk from BLV-infected cattle [9, 58, 59]. BLV proviral DNA has also been detected in row meat and milk products for human consumption [4], indicating that fresh milk and raw meet might be the most likely means of BLV transmission from cows to human [31]. Indeed, a previous study showed a significant association of the consumption of dairy products and beef with breast cancer [60]. Further, evidence for an association between working in the meat industry and increased cancer risk, particularly for lung cancer and hematologic cancer, has been demonstrated [61–63]. In contrast, if BLV incorporates into human from cooked beef and milk, it could not cause infection in human. Japan is one of the countries where the average milk and meat consumption is lower than in Western countries [60]. Japanese traditional food is rich in minimally processed, fresh, seasonal food; fish and vegetables are the main part of the diet, whereas pasteurized dairy products, eggs, and meat make up only a small part of the diet.

There are few reasons which explain why BLV proviral DNA and anti-BLV antibodies could not be detected in the current study. First, Japanese human blood and breast cancer tissue samples are truly free from BLV, with a lack of BLV in at least the screened Japanese human samples. Second, even though the short-fragment PCR method applied in this study showed extremely high sensitivity for detecting even a single copy of BLV *env* and *tax* gene in FLK-BLV DNA template, it might be less sensitive in human sample. Third, BLV may not express gp51 or p24 capsid protein in human blood and may not replicate, resulting in an extremely low level of antibodies, which may be undetectable by ELISA and western blotting, or humans are infected but do not develop any immune response against BLV. Fourth, the study subjects might not be representative of the Japanese population as Japanese human blood samples were randomly obtained from healthy individuals without any information regarding possible exposure to BLV, such as work environment and history of consuming raw cow milk or meat. Further, the number of Japanese human breast cancer tissue samples was insufficient. Therefore, additional studies are needed to verify our result using large numbers of human blood and cancer tissue samples from different occupational backgrounds, including slaughterhouse workers, farmers, and dairy factory workers, who are directly in contact with raw cow milk, as well as veterinarians from wider geographical locations within Japan. In conclusion, although the blood, serum, and breast cancer tissue samples included in this study were negative for BLV, there is a need for additional studies including samples from individuals who are in direct contact with cattle to determine whether BLV can be transmitted to humans.

## Conclusions

In this study, we investigated the presence of BLV in clinical samples from Japan including human blood and breast cancer tissue samples. PCR amplification of both short- and long-fragments of BLV provirus in 97 human blood and 23 breast cancer tissue samples showed the absence of BLV in all tested human samples from Japan. Furthermore, serological tests showed no IgM and IgG antibodies in 97 human sera from Japan against BLV antigens. Further, western blot results further confirmed the ELISA result that there were neither IgM nor IgG antibodies interacting with BLV in our test samples. Thus, our immunological tests showed all of 97 human sera from Japan have no antibodies reactive with BLV antigens. Taken together, our results clearly showed no evidence of BLV proviral DNA in human donated blood and breast cancer samples from Japan and antibodies in human sera against BLV.

## Materials and methods

### Experiment material

Ninety-seven donated human blood samples were provided by the Japanese Red Cross Society (Tokyo, Japan). Sera of the same blood specimens, which were separated for testing infectious diseases and for biochemical assays, were also provided by the Japanese Red Cross Society. All sera were originated from volunteer blood donors who were negative for ten tested infectious diseases (Table 1). Surgically removed frozen breast tissue specimens were acquired from 23 patients with breast cancer at Nihon University, school of Medicine, Itabashi hospital (Tokyo, Japan). The breast tissue donors were diagnosed with invasive breast cancer. This study was conducted in accordance with the guidelines proposed by RIKEN (Approval ID: Wako3 27-21 and Wako 30-1). No information was available on the sex, occupation, age, or race/ethnicity of the donors. All donors provided informed consent for the use of their blood and tissue specimens for research. All protocols involving human subjects were reviewed and approved by the Nihon Red Cross review board, the Human Research Ethics Committee of Nihon University, and RIKEN institutional review board.

### Extraction of DNA and quality evaluating

Genomic DNA was extracted from 97 human blood samples, 23 breast cancer tissue samples, and from FLK-BLV cells, which are permanently infected with BLV, using the Wizard Genomic DNA purification kid (Promega Corporation, Tokyo, Japan) according to the manufacturer`s instructions. The extracted DNA was stored at − 20 °C until required.

To evaluate quality of the extracted DNA, we performed PCR amplification for the human KRAS proto-oncogene using primers listed in Additional file 1: Table S1.

### Proviral copy number measured by digital PCR

To check the sensitivity of PCR applied and for use as a positive control, genomic DNA was extracted from fetal lamb kidney cells permanently infected with BLV (FLK-BLV), and the proviral copy numbers of BLV in these cells were measured using droplet digital PCR (Bio-Rad, Tokyo, Japan); as previously described, the BLV copy numbers was determined to be 12,700 copies/μL in FLK-BLV DNA (50 ng/μL) [47]. FLK-BLV DNA with 100, 10, 5 and 1 copies of provirus were used respective PCRs.

### BLV proviral detection by PCR amplification of the long proviral region

A 500-ng human DNA specimen was spiked with a known amount of FLK-BLV DNA (BLV copy numbers of 100, 10, 5 and 1 in each PCR). Long-fragments (0.8 kb ~ 5.4 kb) of the BLV proviral genome were PCR amplified using PrimeSTAR GXL DNA Polymerase (Takara Bio Inc., Kusatsu, Japan) and the primers listed in Additional file 1: Table S1. The reaction mixture (20 μL/sample) contained 1 × of 5 × PrimeSTAR GXL Buffer, 200 μM of 2.5 mM dNTP mix, 1.25 U/20 μL of PrimeSTAR GXL polymerase, and 0.5 μM of each primer (10 μM). The conditions for PCR amplification were as follows: 98 °C for 2 min, followed by 36–40 (40 in longer fragments) cycles of denaturation at 98 °C for 15 s, annealing at 60 °C for 15 s, and extension at 68 °C for 1 min/kb, followed by post-extension (1 cycle of 68 °C for 4 min) and a final hold at 4 °C. The PCR for each sample was performed in tripled. The *KRAS* gene was also used as a housekeeping gene to confirm the validity of DNA and to optimize the amount of input DNA. PCR products for each sample were loaded onto a 0.8% agarose gel and separated by electrophoresis on the basis of size differences. Distilled water was used as no-template DNA-negative control.

### BLV proviral detection by PCR amplification with short proviral region

All samples were tested for presence of the BLV proviral genome using high-sensitive PCR method described previously [47], using the TaqMan Gene Expression Master Mix (Applied biosystems, Tokyo, Japan) with moderate modifications. Six proviral gene fragments, named LTR, *gag*, *pol*, *env*, *tax,* and *tax*-3`LTR were amplified using primers listed in Additional file 1: Table S1. The reaction mixture (20 μL/sample) contained 500 ng of sample DNA (The amount of FLK-BLV template DNA depend on the BLV copy numbers), 0.2 mM each primer and 1X TaqMan Gene Expression Master Mix. The PCR conditions were as follows: 95 °C for 5 min, followed by 60 cycles of denaturation at 95 °C for 15 s, annealing at 60 °C for 15 s, and extension at 72 °C for 30 s (extension at 72 °C for 50 s for *tax*-3′LTR), followed by post-extension (1 cycle of 72 °C for 4 min) and a final hold at 4 °C. PCR reaction of each sample was performed in triplicate. After amplification, the PCR products were separated by electrophoresis on a 3.0% agarose gel based on size differences. Samples were considered positive for the BLV proviral genome region only if PCR results were obtained at least twice from three different reactions for each target gene.

Throughout the laboratory work with initial samples and DNA, special precautions were taken to prevent cross-contamination among test samples and positive control. DNA was extracted in a clean area, DNA-free room, using a special hood with UV light, along with nucleic acid decontamination solutions for preparing the PCR mixture as well as the addition of DNA to the reaction mix; all work with the positive control DNA, including DNA dilution based on copy number and addition of positive control into the PCR tube, was performed in another room.

### ELISA for anti-BLV p24 antibody

The indirect BLV p24 ELISA was performed based on previously described [57]. ELISA plates (Sumitomo Bakelite Co., Ltd., Tokyo, Japan) were coated with 200 ng/well recombinant BLV capsid p24 antigen diluted in 100 μL in 0.1 M NaHCO_3_ coating buffer (pH 9.0). After overnight incubation at 4 °C, coating buffer was removed and wells were washed for four times for 1 min per wash, with 300 μL/well/wash phosphate-buffered saline (PBS)-T buffer. Human serum specimen diluted 1:50 in sample dilution buffer (7.20 mM Na_2_HPO_4_ 12H_2_O, 2.82 mM NaH_2_PO_4_ 2H_2_O, 150.24 mM NaCl, 1% tween 20, and 5% skim milk) were incubated with antigens for 1 h at 37 °C, as primary antibodies. The plates were then washed and incubated at 37 °C for 30 min with HRP-conjugated goat anti-human IgG (diluted as 1/1000) (Abcam PIC., Tokyo, Japan) and goat anti-human IgM (diluted as 1/10,000) (Abcam PIC.), used as secondary antibodies. Finally, 3,3′,5,5′-tetramethylbenzidine substrate (Thermo Fisher Scientific, Rockford, CA) was applied to the plates and reacted with the test sample for 10 min in dark. The reaction was stopped adding 1 N H_2_SO_4_. OD values were measured at 450 nm with the correction at 690 nm using a EnSight™ multimode plate reader (PerkinElmer Inc., Waltham, MA). The plates were blanked on wells containing only the sample dilution buffer. All samples were run in triplicate. During each assay, to check the validity of the experiment procedure, two control systems were used: (i) Samples from two BLV-infected cattle and one cattle with no BLV infection, were used as positive and negative controls, respectively, followed by incubation with HRP-conjugated rabbit anti-bovine IgG (Thermo Fisher Scientific, Waltham, MA) (diluted as 1/2000), as the second antibody. (ii) To validate whether the human secondary antibodies (HRP-conjugated goat anti-human IgG and IgM) are active, we selected HTLV I/II antibody ELISA kit (MyBioScource Inc., San Diego, CA) and performed ELISA with replacement of secondary antibody in the kit with HRP-conjugated goat anti-human IgG and IgM.

### ELISA for anti-BLV gp51 antibody

Two antibody isoforms (IgG and IgM) in human serum were assessed using a commercially available BLV indirect gp51 ELISA kit (JNC, Tokyo, Japan), according to the manufacturer’s instructions. Human serum specimens, diluted in 1:50 with the sample dilution buffer in the kit, were used as primary antibodies, followed by HRP-conjugated goat anti-human IgG (diluted as 1/1000) (Abcam PIC.) or goat anti-human IgM (diluted as 1/10,000) (Abcam PIC.) the secondary antibody. All samples were run triplicate. During each assay, to check the validity of the experiment procedure, we used two controls, as indicated in ELISA for anti-BLV p24 antibody.

### Western blotting assay

FLK-BLV cells and BLV-free FLK cells were lysed for 30 min on ice in 20 mM Tris–HCl (pH 7.4), 300 mM NaCl, 2 mM ethylenediaminetetraacetic acid, and 2% NP40 supplemented with a protease inhibitor cocktail (Roche Diagnostics, Mannheim, Germany). Lysates were mixed with SDS buffer and boiled for 5 min. Protein concentrations were determined with Pierce™ BCA Protein Assay Kit (Thermo Fisher Scientific). The proteins were then diluted in PBS to a final concentration of 10 ug/mL. Equal amounts of protein were electrophoresed by 12% SDS–polyacrylamide gel electrophoresis (SDS-PAGE) and transferred onto a polyvinylidene difluoride membrane (Millipore, Bedford, MA) using a Trans-Blot Turbo apparatus (Bio-Rad, Irvine, CA). After transfer, the membranes were incubated with blocking buffer with 8% skim milk for 30 min at room temperature and then incubated with human serums were diluted 1:50 with PBS with 5% skim milk, overnight at 4 °C. After washing, the membranes were incubated with HRP-conjugated goat anti-human IgG and IgM (1:100; Abcam PIC.) for 1 h. Sera from cattle infected with EBL and from uninfected cattle were used as positive and negative control, respectively, followed by incubation with rabbit anti-bovine IgG (1:4000; Thermo Fisher Scientific). The signals were visualized using SuperSignal™ West Femto Meximum Sensitivity Substrate (Thermo Fisher Scientific). Images were acquired using Chemiluminescent Imaging system (ATTO Corporation, Tokyo, Japan). PBS was used in place of primary antibody to adjust for any non-specific binding of the secondary antibody. Each human serum samples were tested twice on different days.

## Supplementary Information

**Additional file 1: Table S1.** Primers used to detect BLV DNA in human blood and breast cancer tissue samples.

## Data Availability

The datasets used and/or analyzed during the current study are available from the corresponding author on reasonable request.

## Abbreviations

BLV: Bovine leukemia virus
EBL: Enzootic bovine leucosis
HTLV: Human T-cell leukemia virus
LTR: Long terminal repeat
PCR: Polymerase chain reaction
HIV: Human immunodeficiency virus
ELISA: Enzyme-linked immunosorbent assay
HRP: Horseradish peroxidase
OD: Optical density
PBS: Phosphate-buffered saline
