# Supplementary material for: No evidence of bovine leukemia virus proviral DNA and antibodies in human specimens from Japan
Source: Retrovirology. 2022 May 18;19:7. doi: 10.1186/s12977-022-00592-6 (PMC9116711; doi:10.1186/s12977-022-00592-6)
Supplement: Supplementary file 1 — Additional file 1: Table S1. Primers used to detect BLV DNA in human blood and breast cancer tissue samples. [file 12977_2022_592_MOESM1_ESM.dot]

**Additional file 1: Table S1** Primers used to detect BLV DNA in human blood and breast cancer tissue samples

| **Source** | **PCR ID** | **Primer** | | **Location^b^** | **PCR Product length (bp)** | **Ref.** |
| --- | --- | --- | --- | --- | --- | --- |
|  |  | **Name^a^** | **Sequences 5` to 3`** |  |  |  |
| Human | *KRAS* | Kras-F | GGTGGAGTATTTGATAGTGTATTAACC | 10447-10473 | 411 | [49] |
|  |  | Kras-R | GAACATCATGGACCCTGACA | 10857-10838 |  |  |
| BLV short-fragment PCR | LTR | LTR-F | TGGTGGCTAGAATCCCCGTA | 90-109 | 145 | [49] |
|  |  | LTR-R | GGGTCTCAGGAGAAGAACGC | 234-215 |  |  |
|  | *gag*(p24) | gag-F | GACCAAACGGCCCATATGAC | 1151-1170 | 119 | [49] |
|  |  | gag-R | TTGGGCTGAGCTGATTGTTG | 1269-1250 |  |  |
|  | *pol* | pol-F | ACCAGTCCCTACCTACCTTGC | 2977-2997 | 89 | [49] |
|  |  | pol-R | CCTAGAGACCCATTGGAGGTC | 3065-3045 |  |  |
|  | *env* | env-F | ATGGTCACATATGATTGCGAGC | 5138-5159 | 185 | [49] |
|  |  | env-R | TCATATCCCCATATCTCCCAG | 5322-5302 |  |  |
|  | *tax* | tax-F | TGGAACAACTTAGTAACGCATC | 7659-7680 | 208 | [49] |
|  |  | tax-R | GCTCGCCTAGGGGTAGAATAC | 7866-7846 |  |  |
|  | *tax-*3`LTR | M-tax-F | GTATTCTACCCCTAGGCGAGC | 7846-7866 | 453 | This paper |
|  |  | M-LTR-R | TACGGGGATTCTAGCCACCA | 8298-8279 |  |  |
| BLV long-fragment PCR | 5`LTR | LTR-F | TGGTGGCTAGAATCCCCGTA | 90-109 | 1160 | [49] |
|  |  | gag-R | TTGGGCTGAGCTGATTGTTG | 1269-1250 |  |  |
|  | 3`LTR | tax-F | TGGAACAACTTAGTAACGCATC | 7659-7680 | 764 | [49] |
|  |  | LTR-R | GGGTCTCAGGAGAAGAACGC | 8403-8423 |  |  |
|  | LP-1 | LTR-F | TGGTGGCTAGAATCCCCGTA | 90-109 | 2955 | [49] |
|  |  | Pol-R | CCTAGAGACCCATTGGAGGTC | 3065-3045 |  |  |
|  | LP-2 | env-F | ATGGTCACATATGATTGCGAGC | 5138-5159 | 2708 | [49] |
|  |  | tax-R | GCTCGCCTAGGGGTAGAATAC | 7866-7846 |  |  |
|  | LP-3 | Pol-F | ACCAGTCCCTACCTACCTTGC | 3045-3065 | 2257 | [49] |
|  |  | env-R | TCATATCCCCATATCTCCCAG | 5322-5302 |  |  |
|  | LP-4 | gag-F | GACCAAACGGCCCATATGAC | 1151-1170 | 2811 | [49] |
|  |  | pol-R | CCTAGAGACCCATTGGAGGTC | 3065-3045 |  |  |
|  | LP-5 | gag-F | GACCAAACGGCCCATATGAC | 1151-1170 | 4152 | [49] |
|  |  | env-R | TCATATCCCCATATCTCCCAG | 5322-5302 |  |  |
|  | LP-6 | LTR-F | TGGTGGCTAGAATCCCCGTA | 90-109 | 5217 | [49] |
|  |  | env-R | TCATATCCCCATATCTCCCAG | 5322-5302 |  |  |
|  | LP-7 | Pol-F | ACCAGTCCCTACCTACCTTGC | 3045-3065 | 5378 | [49] |
|  |  | LTR-R | GGGTCTCAGGAGAAGAACGC | 8403-8423 |  |  |

^a^ F, forward primer; R, reverse primer. Reverse sequences are reversed and complementary to the　published reference sequence.

^b^ Location indicates the position within the nucleotide sequence of the *KRAS* proto-oncogene (DDBL: NG_007524 [human]) and the FLK-BLV subclone pBLV913 (DDBL: EF600696 [BLV]).
